# Assessment of corneal biomechanics in anisometropia using Scheimpflug technology

**DOI:** 10.3389/fbioe.2022.994353

**Published:** 2022-10-04

**Authors:** Rongrong Gao, Yuecheng Ren, Siheng Li, Huilin Xu, Xuanqiao Lin, Colm McAlinden, Junming Ye, Jinhai Huang, Jinjin Yu

**Affiliations:** ^1^ School of Ophthalmology and Optometry and Eye Hospital, Wenzhou Medical University, Wenzhou, Zhejiang, China; ^2^ Eye Institute and Department of Ophthalmology, Eye & ENT Hospital, Fudan University, Key Laboratory of Myopia, Chinese Academy of Medical Sciences, Shanghai, China; ^3^ Department of Ophthalmology, Singleton Hospital, Swansea Bay University Health Board, Swansea, United Kingdom; ^4^ Department of Ophthalmology, Royal Gwent Hospital, Aneurin Bevan University Health Board, Newport, United Kingdom; ^5^ Department Ophthalmology, Yiwu Central Hospital, Yiwu, Zhejiang, China; ^6^ Shanghai Research Center of Ophthalmology and Optometry, Shanghai, China

**Keywords:** anisometropia, refractive error, myopia, corneal biomechanics, Scheimpflug technology, Corvis ST

## Abstract

**Purpose:** To investigate the relationship between corneal biomechanical and ocular biometric parameters, and to explore biomechanical asymmetry between anisometropic eyes using the corneal visualization Scheimpflug technology device (Corvis ST).

**Methods:** 180 anisometropic participants were included. Participants were divided into low (1.00≤△Spherical equivalent (SE) < 2.00D), moderate (2.00D≤△SE < 3.00D) and high (△SE ≥ 3.00D) anisometropic groups. Axial length (AL), keratometry, anterior chamber depth (ACD) and corneal biomechanical parameters were assessed using the OA-2000 biometer, Pentacam HR and Corvis ST, respectively.

**Results:** The mean age of participants was 16.09 ± 5.64 years. Stress-Strain Index (SSI) was positively correlated with SE (r = 0.501, *p* < 0.001) and negatively correlated with AL (r = -0.436, *p* < 0.001). Some other Corvis ST parameters had weak correlation with SE or AL. Corneal biomechanical parameters except for time of first applanation (A1T), length of second applanation (A2L), deformation amplitude (DA), first applanation stiffness parameter (SPA1) and ambrosia relational thickness-horizontal (ARTh) were correlated with ametropic parameters (SE or AL) in multiple regression analyses. A1T, velocity of first applanation (A1V), time of second applanation (A2T), A2L, velocity of second applanation (A2V), corneal curvature radius at highest concavity (HCR), peak distance (PD), DA, deformation amplitude ratio max (2 mm) (DAR), SPA1, integrated radius (IR), and SSI showed significant differences between fellow eyes (*p* < 0.05). There was no significant difference in asymmetry of corneal biomechanics among the three groups (*p* > 0.05). Asymmetry of some biomechanical parameters had weak correlation with asymmetry of mean corneal curvatures and ACD. However, asymmetry of corneal biomechanical parameters was not correlated with asymmetry of SE or AL (*p* > 0.05).

**Conclusion:** More myopic eyes had weaker biomechanical properties than the contralateral eye in anisometropia. However, a certain linear relationship between anisometropia and biomechanical asymmetry was not found.

## Introduction

Anisometropia is a significant difference of 1.00D or more in refractive error between the eyes (Vincent et al., 2014). The prevalence of anisometropia was 5.3% in a Chinese elementary schoolchildren population and the prevalence and severity of anisometropia increased with refractive error (Lee C. et al., 2017). Anisometropia can affect binocular function and stereopsis (Birch et al., 2019; Atchison et al., 2020; Singh et al., 2021). In particular, anisometropia in early childhood can also lead to amblyopia (Smith et al., 2017). However, the mechanism of anisometropia has been not clear. Mechanical factors may play important roles in anisometropia (Vincent et al., 2014). The cornea, a crucial part of the ocular optical system, has complex biomechanical properties such as anisotropy, nonlinearity, and viscoelasticity (Esporcatte et al., 2020; Baptista et al., 2021; Chong and Dupps, 2021).

Corneal biomechanics is one of the factors that potentially affects corneal response to orthokeratology (González-Méijome et al., 2008; Wu et al., 2021). The viscoelasticity of the cornea was not only associated with the response but also the recovery of orthokeratology (González-Méijome et al., 2008). What’s more, corneal refractive surgery would change corneal biomechanics and even might lead to corneal ectasia (MA et al., 2016; H et al., 2019) and the corneal biomechanical parameters can predict postoperative refractive error (Wang et al., 2018). Thus, a better understanding of corneal biomechanics in anisometropia will be useful in clinical practice.

At present, there have been lots of studies focused on corneal biomechanical properties for different refractive states of eyes from different individuals (Lee et al., 2016; Long et al., 2019; Sedaghat et al., 2020; Tubtimthong et al., 2020). Multiple confounding factors are implicated in corneal biomechanical measurements *in vivo*. Researchers tried to minimize confounding factors such as age, gender, intraocular pressure (IOP), and central corneal thickness (CCT) and found corneal biomechanical properties are impaired with increasing myopia (Sedaghat et al., 2020; Yu et al., 2020; Ma et al., 2021). However, several factors such as hormonal levels, environmental conditions and corneal hydration status are still hard to eliminate among individuals (Kling and Hafezi, 2017). Anisometropia has fellow eyes with different refractive conditions from the same individual, which are ideal objects to carry out control studies minimizing confounding factors.

Previous studies using the ocular response analyzer (ORA) found that more myopic eyes had lower corneal hysteresis (CH) compared with the fellow eyes in high anisometropia, indicating more myopic eyes had weaker corneal biomechanical properties (Xu et al., 2010). ORA, the first clinical device to measure corneal biomechanics has some limitations, for example, the real meaning of ORA parameters is not clear and CH does not have a direct relationship with stiffness parameters (such as Young’s modulus) (Baptista et al., 2021). The corneal visualization Scheimpflug technology device (Corvis ST), using dynamic Scheimpflug imaging technology can display the dynamic process of corneal deformation under external force in real-time (Esporcatte et al., 2020), maybe a better choice to evaluate corneal biomechanics *in vivo* (Pinero and Alcon, 2015; Jędzierowska and Koprowski, 2019). Its continuously updated parameters help us to assess the corneal biomechanical properties more comprehensively. To the best of our knowledge, there is a lack of studies comparing the corneal biomechanical properties of anisometropia eyes using the Corvis ST.

The purpose of the current study was to investigate the relationship between corneal biomechanical parameters and ocular biometrics in anisometropia with the Corvis ST, and to explore biomechanical asymmetry between anisometropic fellow eyes.

## Materials and methods

### Study population

The study was conducted under the Declaration of Helsinki and approved by the Ethics Committee of the Eye Hospital at Wenzhou Medical University (2021–012-K-09). This was a cross-sectional study. A total of 180 anisometropic patients in the Department of optometry or refractive surgery center of Eye Hospital of Wenzhou Medical University from January 2021 to January 2022 participated in this study. All patients signed and informed consent form.

In our study, anisometropic patients over 10 years old were included. Subjects with any of the following conditions will be excluded: 1) History of corneal surgery (such as refractive surgery, pterygium excision, *etc.*), 2) History of intraocular surgery, 3) History of ocular trauma, 4) Suffering from corneal or eye diseases (such as keratoconus, corneal ulcer, corneal ectasia, glaucoma, *etc.*), 5) Suffering from systemic diseases that affect corneal biomechanics (such as diabetes, connective tissue disease, *etc.*), 6) Pregnant or in menstruation, 7) Recently use of drugs that affect corneal biomechanics (such as prostaglandins), 8) Recently wearing of contact lenses (soft contact lens within 1 week; RGP lens within 1 month; orthokeratology within 3 months), 9) Poor fixation, (10) Accommodative dysfunction. According to the degree of anisometropia, subjects were divided into three subgroups: 1) low anisometropia group: 1.00≤△Spherical equivalent (SE) < 2.00D, 2) moderate anisometropia group: 2.00D≤△SE < 3.00D, 3) high anisometropia group: △SE ≥ 3.00D (asymmetry of parameters were calculated by relative hyperopic value minus relative myopic value and “△” represented binocular asymmetry).

### Methods

Subjects underwent routine eye examination, including subjective refraction, best-corrected visual acuity, slit-lamp and fundus examination. Axial lengths (AL) were measured with OA-2000 biometer (Tomey, Nagoya, Japan). Anterior segment parameters including corneal curvatures and anterior chamber depth (ACD) were measured with the Pentacam HR (Oculus, Wetzlar, Germany). Mean corneal curvatures (Km) were used for analysis. Corneal biomechanical parameters were obtained from the Corvis ST (Oculus, Wetzlar, Germany, 1.6r2187). To avoid corneal deformation affecting the measurement accuracy, the Corvis ST measurement was performed last in each patient. Both eyes of all participants were measured three times using the Corvis ST respectively, with the interval between the adjacent measurements longer than 1 min to restore the cornea to normal. The first eye to be measured was determined randomly. Only measurements with an “OK” quality were used for analysis. If the quality did not achieve an “OK” assessment, the examination was repeated. If the quality of multiple attempts did not achieve an “OK” assessment, the subject was excluded. All examinations were performed by an experienced ophthalmologist.

### Corvis ST and SSI

The Corvis ST consists of a non-contact intraocular pressure measuring instrument and an ultra-high-speed Scheimpflug camera, which can display the dynamic process of corneal deformation under an external force in real-time. After every measurement, a series of corneal biomechanical parameters are generated. The dynamic corneal response parameters (DCR) consist of first applanation (A1) parameters, second applanation (A2) parameters, highest concavity (HC) parameters, Vinciguerra screening parameters. Supplementary Table S1 provides more detail about each parameter.

Stress-Strain Index (SSI) is a new Corvis ST parameter, based on finite element models. SSI is estimated according to numerical modeling with SP-HC, bIOP, and central corneal thickness (CCT) (Eliasy et al., 2019). Different from other parameters, SSI is a corneal stiffness index that can estimate corneal stiffness independent of bIOP and CCT (Eliasy et al., 2019; Liu et al., 2020).

### Statistical analysis

Descriptive data were analyzed using SPSS software (IBM SPSS Statistics for Windows, Version 25.0.0, IBM, Armonk, New York, USA). First, one eye of participants was selected randomly to test the correlation between the corneal biomechanical parameters and ocular biometric parameters using Pearson correlation coefficients. Multiple linear regression with the stepwise method was applied to assess the relationship between corneal biomechanical parameters with SE, AL, ACD, Km, biomechanically corrected intraocular pressure (bIOP), CCT, age and gender. Second, a two-tailed paired *t*-test was used to compare corneal biomechanical properties of the binocular eyes. An analysis of variance (ANOVA) was used to compare corneal biomechanical asymmetry among different anisometropia groups. Pearson correlation coefficients were used to explore correlations between the degree of anisometropia and the asymmetry of corneal biomechanics. A *p*-value less than 0.05 was considered statistically significant.

## Results

180 anisometropic participants were included, 95 (52.8%) males. The mean (± standard deviation) age was 16.09 ± 5.64 years (range: 10–38 years).

### Correlation between corneal biomechanical parameters and ocular biometric parameters

**FIGURE 1:**
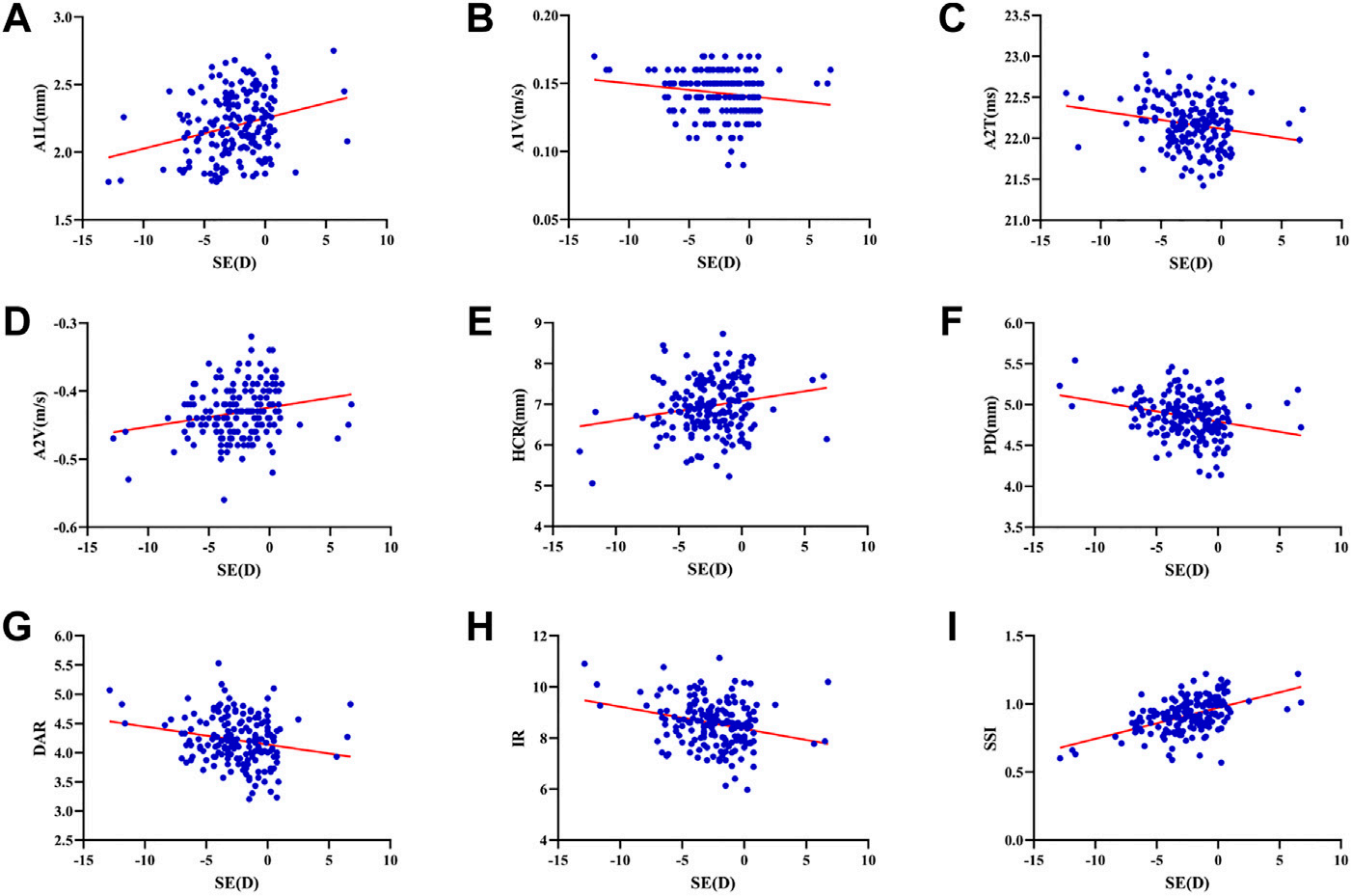
Corneal biomechanical parameters were significantly correlated with the refractive error. SE had significant correlations with A1L (*r* = 0.260, *p* < 0.001) **(A)**, A1V (*r* = -0.174, *p* = 0.020) **(B)**, A2T (*r* = -0.201, *p* = 0.007) **(C)**, A2V (*r* = 0.208, *p* = 0.005) **(D)**, HCR (*r* = 0.198, *p* = 0.008) **(E)**, PD (*r* = -0.271, *p* < 0.001) **(F)**, DAR (*r* = -0.206, *p* = 0.006) **(G)**, IR (*r* = -0.264, *p* < 0.001) **(H)** and SSI (*r* = 0.501, *p* < 0.001) **(I)** (Panel 1A–I).

**FIGURE 2:**
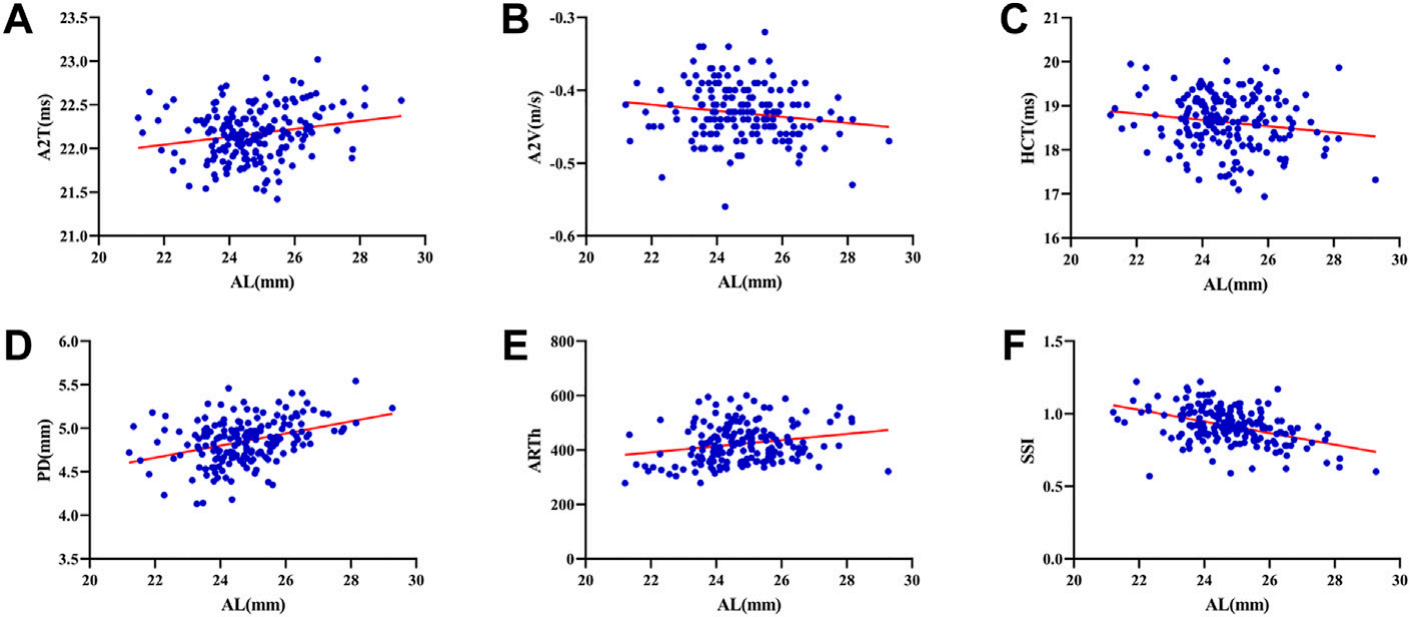
Corneal biomechanical parameters were significantly correlated with the refractive error. AL was significantly correlated with A2T (*r* = 0.205, *p* = 0.006) **(A)**, A2V (*r* = -0.152, *p* = 0.041) **(B)**, HCT (*r* = -0.154 *p* = 0.039) **(C)**, PD (*r* = 0.371, *p* < 0.001) **(D)**, ARTh (*r* = 0.215, *p* = 0.004) **(E)** and SSI (*r* = -0.436, *p* < 0.001) **(F)** (Panel 2A–F).

The mean SE, AL, ACD and Km were -2.44 ± 2.72D (range: 12.88D∼6.75D), 24.75 ± 1.34 mm (range: 21.21–29.27 mm), 3.29 ± 0.30 mm (range: 2.47–4.25 mm), and 42.97 ± 1.38D (range: 39.70D∼47.38D). SE had significant correlations with A1L (*r* = 0.260, *p* < 0.001), A1V (*r* = -0.174, *p* = 0.020), A2T (*r* = -0.201, *p* = 0.007), A2V (*r* = 0.208, *p* = 0.005), HCR (*r* = 0.198, *p* = 0.008), PD (*r* = -0.271, *p* < 0.001), DAR (*r* = -0.206, *p* = 0.006), IR (*r* = -0.264, *p* < 0.001) and SSI (*r* = 0.501, *p* < 0.001) (Figures 1A–I). AL was significantly correlated with A2T (*r* = 0.205, *p* = 0.006), A2V (*r* = -0.152, *p* = 0.041), HCT (*r* = -0.154 *p* = 0.039), PD (*r* = 0.371, *p* < 0.001), ARTh (*r* = 0.215, *p* = 0.004) and SSI (*r* = -0.436, *p* < 0.001) (Figures 2A–F). The correlations between corneal biomechanical parameters and other ocular parameters are displayed in Table 1.

**TABLE 1:** Correlations between corneal biomechanical parameters and ocular biometric parameters.

Parameters	SE (D)		AL (mm)		ACD (mm)		Km (D)		bIOP (mmHg)		CCT (μm)		Age (years)	
	*r*	*p*	*r*	*p*	*r*	*p*	*r*	*p*	*r*	*p*	*r*	*p*	*r*	*p*
A1T (ms)	0.014	0.848	-0.011	0.886	0.106	0.158	0.059	0.433	0.823	**<0.001**	0.405	**<0.001**	-0.234	**0.002**
A1L (mm)	0.260	**<0.001**	-0.123	0.099	0.075	0.319	-0.168	**0.024**	0.266	**<0.001**	0.413	**<0.001**	-0.282	**<0.001**
A1V (m/s)	-0.174	**0.020**	0.108	0.149	-0.115	0.126	0.113	0.131	-0.759	**<0.001**	-0.326	**<0.001**	0.284	**<0.001**
A2T (ms)	-0.201	**0.007**	0.205	**0.006**	-0.086	0.250	-0.046	0.538	-0.542	**<0.001**	0.010	0.891	0.332	**<0.001**
A2L (mm)	0.005	0.951	0.014	0.851	-0.032	0.669	-0.128	0.086	-0.072	0.337	0.152	**0.041**	-0.030	0.685
A2V (m/s)	0.208	**0.005**	-0.152	**0.041**	0.063	0.401	-0.029	0.699	0.679	**<0.001**	0.274	**<0.001**	-0.268	**<0.001**
HCT (ms)	0.139	0.062	-0.154	**0.039**	-0.072	0.338	0.061	0.413	-0.298	**<0.001**	-0.043	0.564	0.092	0.218
HCR (mm)	0.198	**0.008**	-0.040	0.598	-0.033	0.660	-0.371	**<0.001**	0.149	**0.045**	0.442	**<0.001**	0.056	0.456
PD (mm)	-0.271	**<0.001**	0.371	**<0.001**	0.005	0.946	-0.278	**<0.001**	-0.714	**<0.001**	-0.265	**<0.001**	0.338	**<0.001**
DA (mm)	-0.107	0.151	0.084	0.265	-0.076	0.313	0.054	0.468	-0.731	**<0.001**	-0.290	**<0.001**	0.246	**0.001**
DAR	-0.206	**0.006**	0.053	0.479	-0.077	0.307	0.298	**<0.001**	-0.501	**<0.001**	-0.648	**<0.001**	0.252	**0.001**
ARTh	-0.036	0.629	0.215	**0.004**	0.275	**<0.001**	-0.339	**<0.001**	0.028	0.708	0.514	**<0.001**	-0.038	0.611
SPA1	-0.044	0.562	0.089	0.234	0.116	0.121	-0.132	0.078	0.574	**<0.001**	0.678	**<0.001**	-0.096	0.198
IR	-0.264	**<0.001**	0.116	0.121	-0.025	0.736	0.292	**<0.001**	-0.386	**<0.001**	-0.530	**<0.001**	0.142	0.057
SSI	0.501	**<0.001**	-0.436	**<0.001**	-0.174	**0.019**	-0.156	**0.037**	0.119	0.111	0.008	0.919	-0.093	0.216
CBI	0.076	0.312	-0.099	0.184	-0.084	0.261	0.225	**0.002**	-0.278	**<0.001**	-0.612	**<0.001**	0.094	0.207

Bold values indicate *p* <0.05.

### Multiple regression analysis for variables predicting corneal biomechanical parameters

Table 2 displays the regression equation for multivariate regression analysis. Other than A1T, A2L, DA, SP and ARTh, corneal biomechanical parameters had a correlation with ametropic parameters (SE or AL). CCT and bIOP had a greater impact on most corneal biomechanical parameters with larger Standardized β coefficients than the ametropic parameters. However, SSI was only correlated with SE (Standardized β coefficient was 0.501).

**TABLE 2:** Multiple regression analysis for variables predicting corneal biomechanical parameters.

Parameters	Predictors	Unstandardized coefficient β	Standardized coefficient β	*p*-value	Adjusted *R* ^2^
A1T (ms)	Constant	3.758		<0.001	0.822
	bIOP	0.112	0.812	<0.001	
	CCT	0.003	0.382	<0.001	
A1L (mm)	Constant	-0.897		0.094	0.300
	CCT	0.003	0.382	<0.001	
	bIOP	0.029	0.260	<0.001	
	SE	0.041	0.471	<0.001	
	AL	0.048	0.275	0.017	
A1V (m/s)	Constant	0.145		<0.001	0.714
	bIOP	-0.006	-0.774	<0.001	
	CCT	<0.001	-0.271	<0.001	
	Km	0.003	0.238	<0.001	
	AL	0.002	0.165	<0.001	
A2T (ms)	Constant	23.345		<0.001	0.323
	bIOP	-0.076	-0.539	<0.001	
	SE	-0.021	-0.191	0.002	
A2L (mm)	Constant	0.536		0.140	0.018
	CCT	0.001	0.152	0.041	
A2V (m/s)	Constant	−0.773		<0.001	0.552
	bIOP	0.012	0.668	<0.001	
	CCT	<0.001	0.247	<0.001	
	SE	0.003	0.186	<0.001	
HCT (ms)	Constant	22.022		<0.001	0.107
	bIOP	-0.091	-0.306	<0.001	
	AL	-0.078	-0.169	0.018	
HCR (mm)	Constant	13.452		<0.001	0.376
	CCT	0.008	0.379	<0.001	
	Km	-0.215	-0.447	<0.001	
	bIOP	0.079	0.250	<0.001	
	AL	-0.131	−0.266	<0.001	
	Age	0.026	0.219	0.001	
PD (mm)	Constant	6.860		<0.001	0.690
	bIOP	-0.082	-0.676	<0.001	
	AL	0.056	0.299	<0.001	
	CCT	-0.002	-0.269	<0.001	
	Km	-0.021	-0.115	0.013	
DA (mm)	Constant	1.569		<0.001	0.631
	bIOP	-0.034	-0.743	<0.001	
	CCT	-0.001	-0.261	<0.001	
	Gender	0.027	0.146	0.002	
	Km	0.010	0.148	0.002	
DAR	Constant	4.311		<0.001	0.745
	CCT	-0.007	-0.584	<0.001	
	bIOP	-0.102	-0.521	<0.001	
	Km	0.101	0.342	<0.001	
	AL	0.051	0.168	<0.001	
SP	Constant	-90.458		<0.001	0.784
	CCT	0.329	0.647	<0.001	
	bIOP	4.764	0.602	<0.001	
	Km	-1.363	-0.114	0.002	
	Age	0.303	0.104	0.005	
ARTh	Constant	205.200		0.191	0.375
	CCT	1.025	0.469	<0.001	
	Km	-12.332	-0.240	<0.001	
	ACD	54.646	0.230	<0.001	
IR	Constant	4.252		0.117	0.530
	CCT	-0.013	-0.467	<0.001	
	bIOP	-0.176	-0.409	<0.001	
	Km	0.239	0.368	<0.001	
	AL	0.162	0.244	<0.001	
SSI	Constant	0.972		<0.001	0.247
	SE	0.023	0.501	<0.001	
CBI	Constant	2.582		<0.001	0.475
	CCT	-0.004	-0.582	<0.001	
	bIOP	-0.031	-0.285	<0.001	
	AL	-0.022	-0.128	0.030	
	Km	0.021	0.16	0.036	

### Comparison of ocular biometric and corneal biomechanical parameters between anisometropic fellow eyes

**FIGURE 3:**
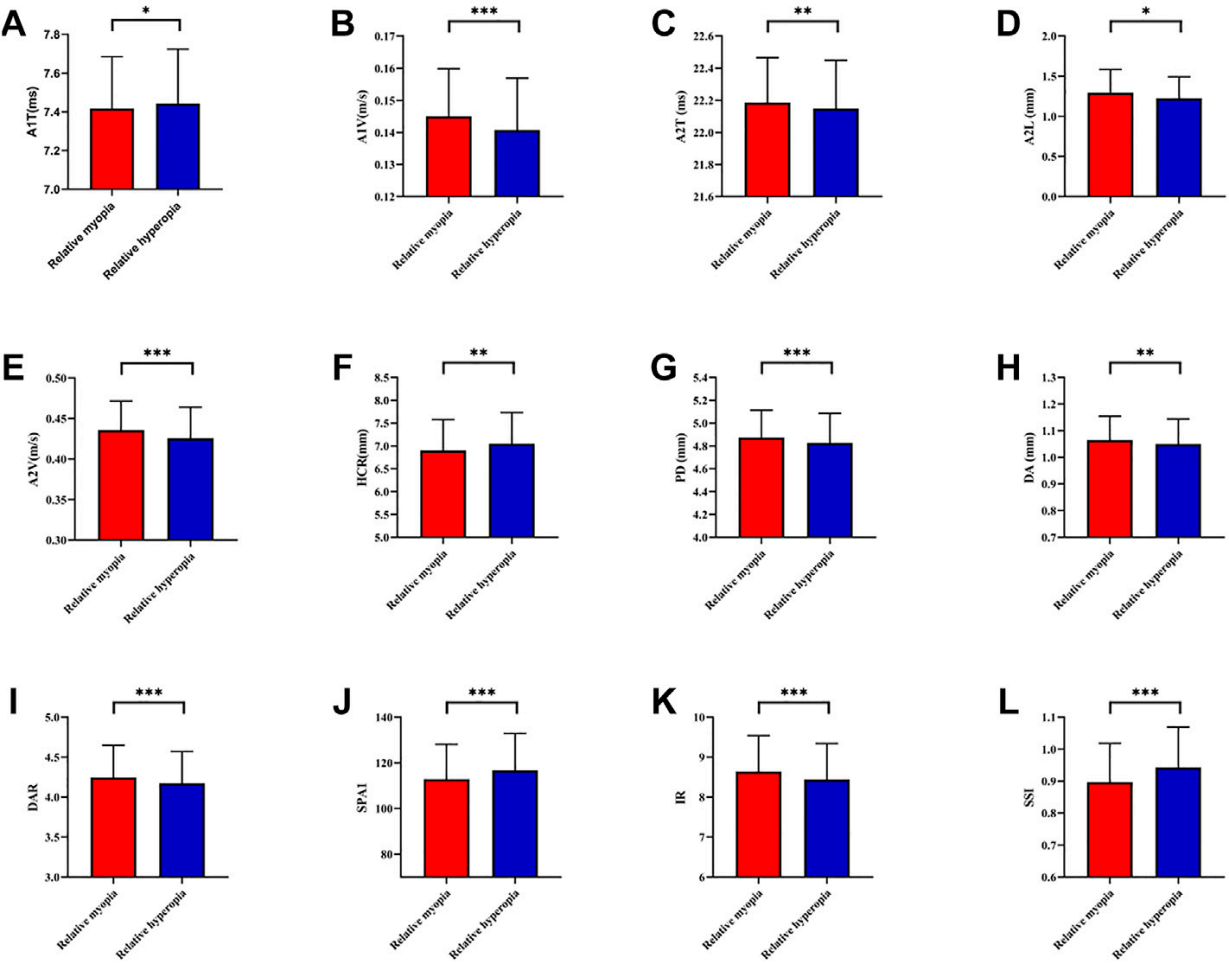
Histogram comparison of A1T, A1V, A2T, A2L, A2V, HCR, PD, DA, DAR, SPA1, IR, and SSI **(A–L)** in anisometropia fellow eyes. *means *p* < 0.05, **means *p* < 0.01 ***means *p* < 0.001.

The SE was -3.52 ± 2.16D in relative myopic eyes and -0.92 ± 2.10D in the contralateral eyes. The mean ∆ SE was 2.53 ± 1.16D. There were statistically significant differences between fellow eyes for refractive error, AL and other ocular biometric parameters (*p* < 0.05) but not for best-corrected visual acuity (*p* = 0.840) (Table 3). The relative myopic eyes had greater A1V, A2T, A2L, A2V, PD, DA, DAR, IR, and smaller A1T, HCR, SPA1, and SSI than contralateral eyes (*p* < 0.05) (Table 4) (Figures 3A–L).

**TABLE 3:** Ocular biometric parameters for anisometropic fellow eyes.

Ocular biometric parameters	Relative myopic eyes	Relative hyperopic eyes	*p*-value
Subjective refraction sphere (D)	-3.52 ± 2.16	-0.92 ± 2.10	<**0.001**
Subjective refraction cylinder (D)	-0.44 ± 0.56	-0.58 ± 0.64	<**0.001**
Spherical equivalent (D)	-3.74 ± 2.28	-1.21 ± 2.17	<**0.001**
Best corrected visual acuity (logMAR)	-0.02 ± 0.09	-0.02 ± 0.06	0.840
Axial length (mm)	25.31 ± 1.16	24.22 ± 1.15	<**0.001**
Anterior chamber depth (mm)	3.31 ± 0.28	3.26 ± 0.30	<**0.001**
Mean keratometry (D)	43.00 ± 1.38	42.94 ± 1.39	**0.031**
Central corneal thickness (μm)	553.11 ± 32.19	554.34 ± 32.33	**0.022**
bIOP for Corvis ST (mmHg)	15.91 ± 1.90	16.16 ± 2.08	**0.005**

Bold values indicate *p* <0.05.

**TABLE 4:** Comparisons in corneal biomechanical parameters between anisometropic fellow eyes.

Biomechanical parameters	Relative myopic eyes	Relative hyperopic eyes	*p*-value
A1T (m/s)	7.42 ± 0.27	7.44 ± 0.28	**0.030**
A1L (mm)	2.21 ± 0.23	2.21 ± 0.24	0.690
A1V (m/s)	0.14 ± 0.01	0.14 ± 0.02	**<0.001**
A2T (ms)	22.19 ± 0.28	22.15 ± 0.30	**0.003**
A2L (mm)	1.30 ± 0.29	1.23 ± 0.27	**0.010**
A2V (m/s)	-0.44 ± 0.04	-0.43 ± 0.04	**<0.001**
HCT (ms)	18.63 ± 0.65	18.69 ± 0.63	0.285
HCR (mm)	6.90 ± 0.67	7.05 ± 0.68	**0.005**
PD (mm)	4.87 ± 0.24	4.83 ± 0.26	**<0.001**
DA (mm)	1.07 ± 0.09	1.05 ± 0.09	**0.001**
DAR	4.25 ± 0.40	4.17 ± 0.40	**<0.001**
ARTh	425.11 ± 69.93	420.01 ± 75.38	0.153
SPA1	112.89 ± 15.24	116.70 ± 16.23	**<0.001**
IR	8.64 ± 0.90	8.44 ± 0.89	**<0.001**
SSI	0.90 ± 0.12	0.94 ± 0.13	**<0.001**
CBI	0.19 ± 0.23	0.19 ± 0.23	0.787

Bold values indicate *p* <0.05.

### Comparison of ocular biometric and corneal biomechanical asymmetry in varying severities of anisometropia

The number of participants in low, moderate and high anisometropia were 61 (33.5%), 61 (33.5%), 58 (32.2%), respectively. Mean ∆SE of three groups were 1.41 ± 0.26D, 2.37 ± 0.29D and 3.89 ± 0.89D. There were no statistically significant differences regarding the age, gender, mean SE or AL of fellow eyes among three groups (*p* > 0.05). More details were shown in Table 5. There were significant differences in asymmetry of SE, AL and ACD (*p* < 0.05) but not in corneal biomechanical asymmetry (*p* > 0.05) between anisometropic fellow eyes among three groups (Supplementary Table S2).

**TABLE 5:** Participant demographics among three groups.

Parameters	Low anisometropia	Moderate anisometropia	High anisometropia	*p*-value
Number (%)	61 (33.5%)	61 (33.5%)	58 (32.2%)	-
Gender (Female/Male)	26/35	31/30	28/30	0.650
Age (years)	15.02 ± 4.21	16.15 ± 6.31	17.17 ± 6.07	0.113
Asymmetry of spherical equivalent (D)	1.41 ± 0.26	2.37 ± 0.29	3.89 ± 0.89	**<0.001**
Spherical equivalent of relative myopia eyes (D)	-2.91 ± 1.99	-3.71 ± 2.37	-4.65 ± 2.16	**<0.001**
Axial length of relative myopia eyes (mm)	25.15 ± 0.98	25.19 ± 1.26	25.60 ± 1.21	0.073
Spherical equivalent of relative hyperopia eyes (D)	-1.50 ± 2.00	-1.34 ± 2.41	-0.77 ± 2.05	0.155
Axial length of relative hyperopia eyes (mm)	24.52 ± 0.97	24.17 ± 1.28	23.96 ± 1.12	**0.025**
Mean spherical equivalent of fellow eyes (D)	-2.21 ± 1.99	-2.52 ± 2.39	-2.71 ± 2.06	0.438
Mean axial length of fellow eyes (mm)	24.84 ± 0.97	24.68 ± 1.26	24.78 ± 1.15	0.754

Bold values indicate *p* <0.05.

### Correlation between corneal biomechanical asymmetry and degrees of anisometropia

△ACD was statistically significant correlated with △HCT (*r* = 0.161, *p* = 0.031) and △CBI (*r* = 0.200, *p* = 0.007). △Km was negatively associated with △SSI (*r* = -0.153, *p* = 0.041). There was no significant difference between the asymmetry of corneal biomechanical parameters and △SE or △AL (*p* > 0.05). More details showed in Supplementary Table S3.

## Discussion

This was the first study to evaluate corneal biomechanics in anisometropia using the Corvis ST. The current study found that several corneal biomechanical parameters based on Corvis ST were slightly correlated with SE or AL (Table 1 and Figure 1 and Figure 2). PD was negatively correlated with SE (Figure 1F) and positively correlated with AL (Figure 2D). HCR was positively correlated with SE (Figure 1E). These findings were in agreement with those from Sedaghat et al. (Sedaghat et al., 2020), Lu et al. (Lu et al., 2022) and Wang et al. (Wang et al., 2015). Deformable parameters (HC parameters) indicated the elastic property of corneal collagen fibers (He et al., 2017). A smaller HCR and higher PD are associated with less resistance to deformation (Long et al., 2019; Esporcatte et al., 2020). We also found DAR and IR were negatively correlated with SE (Figures 1G,H), and positively correlated with AL in the regression analysis (Table 2). Kenia et al. (Kenia et al., 2020) found similar correlations between DAR, IR and SE. Vinciguerra screening parameters are a series of newly developed parameters based on an attempt to differentiate normal corneas from keratoconus corneas (Han et al., 2021). Smaller DAR and IR values indicated a softer cornea (Han et al., 2021). However, CCT and bIOP had a greater impact on most corneal deformation parameters (Table 2), which was similar to previous studies (Sedaghat et al., 2020; Yu et al., 2020). There have been remained a challenge that it is difficult to separate corneal biomechanical parameters from the effects of IOP and CCT *in vivo* measurement (Lee H. et al., 2017).

SSI is a new parameter SSI free of the influences of IOP and CCT (either IOP or CCT was not correlated with SSI (*p* > 0.05)) (Eliasy et al., 2019; Liu et al., 2020). We found SSI had no correlation with CCT and bIOP (Tables 1, 2). However, SSI was weakly correlated with bIOP (*r* = 0.23, *p* < 0.01) in another study (Han et al., 2020a). Although the results in different studies were not consistent, they also indicated SSI reduced the influence of bIOP and CCT on corneal biomechanical measurement. What’s more, SSI is the first standard mechanic metric *in vivo* which provides the whole stress-strain curve (Esporcatte et al., 2020), regardless of the level of the load (Eliasy et al., 2019). It helps us to assess cornea (a nonlinear viscoelasticity tissue) biomechanics better (Kling and Hafezi, 2017; Chong and Dupps, 2021). The current study indicated that SSI was correlated with SE (*r* = 0.501, *p* < 0.001) (Figure 1I) and AL (*r* = -0.436, *p* < 0.001) (Figure 2F). In line with our study, Liu et al. (Liu et al., 2021) indicated SSI was negatively correlated with AL (*r* = -0.476, *p* < 0.001). Han et al. (Han et al., 2020b) found SSI had a positive correlation with SE (*r* = 0.313, *p* < 0.01). Compared to other Corvis ST parameters, the result of SSI was more convincing to show that corneal biomechanical properties became weaker with the increase in myopia. High myopia is associated with abnormal scleral collagen fiber orientation and reduced diameter of fibers, which cause scleral biomechanics to weaken (Markov et al., 2018). Both the cornea and sclera derive from the same mesoderm, so the cornea may have similar changes to the sclera with myopia progression (He et al., 2017). A study based on form-deprivation myopia chicks showed myopic corneal tangent modulus became lower (Kang et al., 2018). Another study about the biophysical properties of corneal cells indicated that F-actin and microtubule content was changing with myopia inducing and recovering in the chick model. F-actin and microtubule content may affect corneal biomechanics (Xin et al., 2021). On the other hand, softer sclera in myopia will weaken the restrictive effect on corneal deformation (Sedaghat et al., 2020).

Age is one of the important factors in corneal biomechanics. Similar to previous studies (Celebi et al., 2018; Eliasy et al., 2019; Liu et al., 2020; Baptista et al., 2021), several Corvis ST parameters were correlated with age, indicating the cornea became stiffer with increasing age (Table 1 and Table 2). More glycation-induced (Kling and Hafezi, 2017) and sun-related (Baptista et al., 2021) cross-linking contribute to this change. However, there was no significant correlation between SSI and age (Table 1 and Table 2). Liu et al. found SSI was relatively stable in a young population and increased with age after the age of 35 (Liu et al., 2020). Most participants in our study were young (mean age was 16.09 ± 5.64 years). Thus, there was no correlation between SSI and age in our study.

Compared with traditional controlled research (comparison among emmetropes and various degrees of ametropia), the assessment of corneal biomechanics in anisometropic eyes would be more convenient to control confounding factors, such as hormonal level, environmental factors, and genetic factors. The current study found relative myopic eyes had greater A1V, A2T, A2L, A2V, PD, DA DAR, IR, and smaller A1T, HCR, SPA1, and SSI than contralateral eyes (*p* < 0.05) (Figures 3A–L), indicating corneal biomechanics was weaker in relative myopic eyes. But the difference was slight. Vincent and others did not find any differences in ORA parameters between anisometropic eyes (11.35 ± 1.37 vs. 11.30 ± 1.41, *p* > 0.05; ∆ SE was 1.70 ± 0.74D) (Vincent et al., 2011). However, Xu et al. (Xu et al., 2010) also found more myopic eyes had lower CH than contralateral eyes using ORA in anisometropic participants (10.0 ± 1.6 vs. 11.0 ± 1.4, *p* = 0.035; ∆ SE was 10.82 ± 3.22D). The different results among three studies may attribute to the varying severity of anisometropia ( ∆ SE was 10.82 ± 3.22D vs. 1.70 ± 0.74D vs. 2.53 ± 1.16D). ∆ SE in our study was slightly higher than Vincent et al. Besides, the opponent results may due to the different biomechanical instruments. Corvis ST yields a stable peak pressure in every examination and the Scheimpflug camera can capture details better than the reflection of the infrared beam in ORA (Esporcatte et al., 2020). Our result in anisometropia was consistent with previous studies based on the ametropia population (Zhang et al., 2018; Long et al., 2019; Tubtimthong et al., 2020; Ma et al., 2021). However, studies based on different ametropic individuals did not find significant differences in A1 parameters (Long et al., 2019; Kenia et al., 2020; Yu et al., 2020). It may attribute to the fact that the comparison between the anisometropia is more able to control for the confounders.

There is a hypothesis that increased IOP results in axial elongation and IOP may be one of the potential mechanical factors leading to myopia and anisometropia (Vincent et al., 2014). However, previous studies found IOP was symmetrical in anisometropic eyes using cornea-corrected IOP from the ORA (Xu et al., 2010; Vincent et al., 2011). The bIOP based on the Corvis ST can reduce the effect of corneal stiffness (Maklad et al., 2020). Relative myopic eyes had significant but not clinically meaningful lower bIOP than contralateral eyes in the current study (Table 3). It did not support this hypothesis and indicated that anisometropia developed with similar IOPs.

Scleral biomechanics were closely related to ocular elongation (Markov et al., 2018). It is supposed that scleral biomechanical changes can be transmitted to the cornea (Nguyen et al., 2020). According to this hypothesis, the first part of our study and previous studies found significant correlations between corneal biomechanics and the severity of myopia. However, our result showed this hypothesis may not make sense in anisometropia. In the current study, there was no significant difference of corneal biomechanical asymmetry among three groups (Supplementary Table S2), and corneal biomechanical asymmetry was no significantly correlated with the asymmetry of AL or SE (Supplementary Table S3). There are several possible explanations as follow. Firstly, the participants were healthy without corneal disease such as corneal ectasia, their corneal biomechanical properties were within a healthy range. The primary biometric basis of anisometropia is the asymmetry in axial length which is related to the sclera (Vincent et al., 2014). When anisometropia becomes severe, scleral biomechanical asymmetry is increasing but asymmetry in corneal biomechanics may remain within a normal range. Secondly, several factors play an important role in anisometropia, such as stochastic factors (variability that comes about from randomness or noise) (Flitcroft et al., 2021) and asymmetric visual experience between fellow eyes (Vincent et al., 2014). But the corneal biomechanical asymmetry between anisometropic eyes may be limited by the same genetic background. Thirdly, even though cornea and scleral are a whole, their biomechanical behaviors are still different when stress is applied (Bronte-Ciriza et al., 2021), thus corneal biomechanical measures may not fully represent the scleral biomechanics.

Significant correlations were found between the asymmetry of several corneal deformation parameters and anterior parameters (ACD and Km) (Supplementary Table S3). The anterior chamber will resist deformation when the cornea moves into anterior chamber with the air pulse (Tejwani et al., 2019). Corneal biomechanical measurements reflect anterior rather than whole ocular biomechanics in anisometropia. However, anisometropia is weaker correlated with anterior chamber depth than AL (the Pearson correlation was 0.183 vs. 0.735) (Hashemi et al., 2013), indicating the weak role of anterior segment factors in anisometropia. Longitudinal studies are needed to further observe the impact of anisometropia and myopia progression on biomechanics. Besides, novel *in vivo* devices that directly assess scleral biomechanics is needed to study the biomechanical mechanisms of anisometropia.

In conclusion, the relative myopia eyes had slightly weaker biomechanical properties than the contralateral eye in anisometropia. However, a certain linear relationship between anisometropia and biomechanical asymmetry was not found. Future studies including scleral biomechanics in anisometropia using novel devices are warranted.

## Data Availability

The original contributions presented in the study are included in the article/Supplementary Material, further inquiries can be directed to the corresponding authors.
