# Supplementary material for: Assessment of corneal biomechanics in anisometropia using Scheimpflug technology
Source: Front Bioeng Biotechnol. 2022 Oct 4;10:994353. doi: 10.3389/fbioe.2022.994353 (PMC9632863; doi:10.3389/fbioe.2022.994353)
Supplement: Supplementary file 1 [file DataSheet1.docx]

Table A. Description of Corvis ST Parameters

| Parameter (Abbreviation) | Description in brief |
| --- | --- |
| First applanation (A1) | Moment at the first applanation of cornea by air puff |
| A1 Time (A1T) | Time from start to A1 |
| A1 Length (A1L) | Length of flattened cornea at A1 |
| A1 Velocity (A1V) | Velocity of corneal apex at A1 |
| Second applanation (A2) | Moment at the second applanation of cornea during restore |
| A2 Time (A2T) | Time from start to A2 |
| A2 Length (A2L) | Length of flattened cornea at A2 |
| A2 Velocity (A2V) | Velocity of corneal apex at A2 |
| Highest concavity (HC) | Moment at the cornea reach maximum deformation |
| HC Time (HCT) | Time from start to HC |
| HC Radius (HC Radius) | Radius of cornea curvature at HC |
| Peak distance (PD) | Distance between the two peaks of cornea at HC |
| Deformation amplitude (DA) | Deformation amplitude of cornea apex at HC |
| Vinciguerra parameters |  |
| Deformation amplitude radio (DAR) | Ratio between the DA at the apex and the average DA at 2mm from the center cornea |
| Ambrósio’s relational thickness to the horizontal profile (ARTh) | Corneal thickness progression in the temporal-nasal direction |
| Stiffness parameter at A1 (SPA1) | Corneal stiffness characteristic generated by A1 parameters |
| Integrated radius (IR) | Area under the inverse concave radius versus time curve |
| Corvis biomechanical index (CBI) | The combination of corvis parameters by logistic regression analysis |

Table B. Asymmetry of ocular biometric and corneal biomechanical parameters in varying severities of anisometropia

| Asymmetry of parameters | low anisometropia | moderate anisometropia | high anisometropia | *p*-value |
| --- | --- | --- | --- | --- |
| Spherical equivalent (D) | 1.41 ± 0.26 | 2.37 ± 0.29 | 3.89 ± 0.89 | **<0.001** |
| Axial length (mm) | -0.63 ± 0.22 | -1.02 ± 0.21 | -1.64 ± 0.43 | **<0.001** |
| Anterior chamber depth (mm) | -0.03 ± 0.06 | -0.04 ± 0.11 | -0.07 ± 0.14 | **0.009** |
| Mean keratometry (D) | -0.09 ± 0.31 | -0.04 ± 0.25 | -0.02 ± 0.32 | 0.442 |
| Central corneal thickness (um) | 0.81 ± 7.11 | 2.26 ± 6.72 | 0.59 ± 7.63 | 0.384 |
| bIOP for Corvis ST (mmHg) | 0.30 ± 1.36 | 0.29 ± 1.10 | 0.16 ± 1.10 | 0.768 |
| Bionechanical parameters | | | | |
| A1T (ms) | 0.03 ± 0.16 | 0.03 ± 0.15 | 0.01 ± 0.15 | 0.792 |
| A1L (mm) | 0.03 ± 0.24 | -0.01 ± 0.26 | 0.00 ± 0.26 | 0.743 |
| A1V (m/s) | 0.00 ± 0.01 | 0.00 ± 0.01 | 0.00 ± 0.01 | 0.813 |
| A2T (ms) | -0.08 ± 0.21 | -0.02 ± 0.13 | -0.01 ± 0.15 | 0.080 |
| A2L (mm) | -0.04 ± 0.34 | -0.09 ± 0.37 | -0.09 ± 0.38 | 0.683 |
| A2V (m/s) | 0.01 ± 0.03 | 0.01 ± 0.03 | 0.01 ± 0.02 | 0.441 |
| HCT (ms) | 0.14 ± 0.73 | 0.09 ± 0.75 | -0.06 ± 0.76 | 0.322 |
| HCR (mm) | 0.11 ± 0.73 | 0.13 ± 0.70 | 0.19 ± 0.62 | 0.810 |
| PD (mm) | -0.06± 0.15 | -0.05 ± 0.19 | -0.04 ± 0.12 | 0.836 |
| DA (mm) | -0.02 ± 0.06 | -0.02 ± 0.06 | -0.01 ± 0.06 | 0.633 |
| DAR | -0.09 ± 0.19 | -0.06 ± 0.15 | -0.08 ± 0.20 | 0.678 |
| ARTh | -9.86 ± 49.46 | -1.91 ± 41.41 | -3.46 ± 52.24 | 0.625 |
| SPA1 | 4.10 ± 10.46 | 4.25 ± 8.43 | 3.05 ± 9.86 | 0.760 |
| IR | -0.17 ± 0.67 | -0.23 ± 0.56 | -0.20 ± 0.59 | 0.883 |
| SSI | 0.06 ± 0.08 | 0.04 ± 0.07 | 0.05 ± 0.08 | 0.362 |
| CBI | 0.00 ± 0.14 | -0.01 ± 0.12 | 0.02 ± 0.13 | 0.578 |

Bold values indicate *P*＜0.05

Table C. Correlations between corneal biomechanical asymmetry and degrees of anisometropia

| Parameters | △SE (D) | | △AL (mm) | | △ACD (mm) | | △Km (D) | |
| --- | --- | --- | --- | --- | --- | --- | --- | --- |
|  | *r* | *p* | *r* | *p* | *r* | *p* | *r* | *p* |
| △A1T (ms) | -0.016 | 0.836 | 0.021 | 0.785 | -0.085 | 0.254 | -0.016 | 0.831 |
| △A1L (mm) | -0.052 | 0.489 | 0.091 | 0.224 | -0.088 | 0.238 | -0.102 | 0.173 |
| △A1V (m/s) | 0.023 | 0.761 | 0.001 | 0.986 | 0.044 | 0.554 | 0.006 | 0.939 |
| △A2T (ms) | 0.065 | 0.389 | -0.058 | 0.442 | 0.094 | 0.211 | 0.011 | 0.881 |
| △A2L (mm) | -0.023 | 0.758 | -0.004 | 0.962 | 0.030 | 0.691 | -0.008 | 0.915 |
| △A2V (m/s) | -0.064 | 0.390 | 0.058 | 0.436 | 0.013 | 0.857 | -0.085 | 0.255 |
| △HCT (ms) | -0.063 | 0.400 | 0.108 | 0.148 | 0.161 | **0.031** | -0.099 | 0.186 |
| △HCR (mm) | 0.066 | 0.377 | -0.074 | 0.321 | 0.032 | 0.667 | -0.103 | 0.169 |
| △PD (mm) | -0.004 | 0.961 | 0.007 | 0.931 | 0.067 | 0.370 | 0.003 | 0.972 |
| △DA (mm) | 0.062 | 0.407 | -0.029 | 0.704 | 0.069 | 0.359 | 0.021 | 0.783 |
| △DAR | -0.012 | 0.872 | 0.047 | 0.528 | 0.078 | 0.297 | -0.038 | 0.615 |
| △ARTh | 0.005 | 0.951 | 0.064 | 0.391 | -0.002 | 0.981 | 0.105 | 0.162 |
| △SPA1 | -0.028 | 0.709 | -0.012 | 0.873 | -0.083 | 0.267 | 0.030 | 0.689 |
| △IR | -0.044 | 0.558 | 0.033 | 0.662 | -0.038 | 0.615 | 0.020 | 0.790 |
| △SSI | 0.049 | 0.516 | -0.035 | 0.645 | 0.009 | 0.906 | -0.153 | **0.041** |
| △CBI | 0.080 | 0.286 | -0.107 | 0.152 | 0.200 | **0.007** | 0.017 | 0.821 |

Bold values indicate *P*＜0.05
